# Supplementary material for: Estimates of the prevalence of male circumcision in sub-Saharan Africa from 2010–2023—A systematic review and meta-analysis
Source: PLoS One. 2024 Mar 13;19(3):e0298387. doi: 10.1371/journal.pone.0298387 (PMC10936832; doi:10.1371/journal.pone.0298387)
Supplement: S1 Table — This table shows the characteristics of included studies. (DOCX) [file pone.0298387.s002.docx]

Supplementary Table 1 – Characteristics of included studies

| Study | Study design | Study period | Nationally representative | Period | Participants | Country | Setting | Sample |
| --- | --- | --- | --- | --- | --- | --- | --- | --- |
| Auvert et al. 2013 [18] | cross-sectional study | 2007-2008  2010-2011 | No | 2010-2015 | Men 15-49 | South Africa | Rural | 5336 |
| Kibira et al. 2014 [19] | cross-sectional study | 2011 | No | 2010-2015 | Men 15- 59 | Uganda | Urban | 7969 |
| Ortblad et al. 2018 [20] | Cohort | 2009-2014 | No | 2010-2015 | Men 15-49 | South Africa | Rural | 13022 |
| Rupfutse et al. 2014 [21] | cross-sectional study | 2014 | No | 2010-2015 | Men 18-49 | Zimbabwe | Rural | 300 |
| Baisley et al. 2018 [22] | Cohort | 2011  2015 | No | 2010-2015 | Men 20–29, HIV negative | South Africa | Rural | 2713 |
| Edossa et al. 2020 [23] | cross-sectional study | 2016 | No | 2015-2023 | Men 15–59 | Ethiopia | Urban and rural | 868 |
| Forbes et al. 2012 [24] | cross-sectional study | 2007-2008 | No | 2010-2015 | Men 15-34 | Tanzania | Rural | 7177 |
| Galbraith et al. 2014 [25] | cross-sectional study | 2007  2012 | No | 2010-2015 | men 15-64 | kenya | Urban and rural | 13416 |
| Hensen et al. 2019 [26] | cross-sectional study | 2013 | No | 2010-2015 | Men 15-59 | Zambia | Rural | 2354 |
| Kim et al. 2019 [27] | cross-sectional study | 2011-2012  2015-2016 | Yes | Both | Men 15-49 | Tanzania | Urban | 11866 |
| Kong et al. 2014 [28] | cross-sectional study | 2010-2011 | No | 2010-2015 | Non-Muslim men 15-49 | Uganda | Rural | 5606 |
| Marshall et al. 2017 [29] | prospective interventional study | 2015 | No | 2015-2023 | Men 18-49 | South African | Rural | 522 |
| Mavhu et al. 2011 [30] | cross-sectional study | 2009 | No | 2010-2015 | Men 18-44 | Zimbabwe | Rural | 999 |
| Mutombo et al. 2015 [31] | cross-sectional study | 2010 | Yes | 2010-2015 | Men 15+ | Malawi | Urban and rural | 4358 |
| Nzamwita et al. 2021 [32] | cross-sectional study | 2019 | No | 2015-2023 | Men 18-43 | Rwanda | Urban | 438 |
| Odoyo-June et al. 2017 [33] | cross-sectional study | 2014-2015 | No | 2010-2015 | Men 25-39 | Kenya | Urban | 5639 |
| Odoyo-June et al. 2021 [34] | cross-sectional study | 2019 | No | 2015-2023 | Men 10-29 | Kenya | Urban and rural | 3569 |
| Peltzer et al. 2014 [35] | Cross-sectional study | 2008 | Yes | 2010-2015 | Men 15-49 | South Africa | Urban and rural | 6654 |
| Tapera et al. 2017 [36] | Cross-sectional study | 2016 | No | 2015-2023 | Men 17-25 | Republic of Botswana | Urban | 303 |
| Westercamp et al. 2017 [37] | Cross-sectional study | 2009, 2011, 2013 | No | 2010-2015 | Men 15-49 | Kenya | Urban | 3355 |
| Tram 2014 [38] | cross-sectional study | 2006 - 2011 | Yes | 2010-2015 | Men +15 | 12 countries* | Urban and rural | 65304 |
| Gasasira et al 2012 [39] | cross-sectional study | 2010 | Yes | 2010-2015 | men | Rwanda | Urban and rural | 1098 |
| Shezi et al 2023 [40] | cross-sectional study | 2018 | No | 2015-2023 | men | Eswatini | Urban and rural | 407 |
| Wambura et al 2011 [16] | cross-sectional study | 2011 | No | 2010-2015 | Men and women aged 18-44 | Tanzania | Urban and rural | 170 |
| Nanteza et al 2018 [41] | cross-sectional study | 2015 | No | 2015-2023 | Men | Uganda | Urban and rural | 396 |
| Hatzold et al 2014 [42] | cross-sectional study | 2013 | Yes | 2010-2015 | Men | Zimbabwe | Urban and rural | 1165 |
| Marukutira et al 2022 [43] | pair-matched community-randomized trial | 2013-2016 | Yes | Both | HIV-negative and -unknown men | Botswana | Urban and rural | 12,864 |
| Keetile, M 2020 [44] | cross-sectional study | 2008, 2013 | Yes | Both | Men aged 10-64 | Botswana | Urban and rural | 10077 |
| Zuma et al 2022 [17] | cross-sectional study | 2017 | Yes | 2015-2023 | Persons of all ages living in the selected households | South Africa | Urban and rural | 36628 |
| Hines et al 2021 [45] | cross-sectional study | 2014-2015, 2018-2019 | No | Both | Men aged 15-59 | Mozambique | urban and rural | 2285 |
| DHS 2017 (Malawi) [46] | cross-sectional study | 2015-2016 | Yes | 2015-2023 | Men aged 15-54 | Malawi | Urban and rural | 7,478 |
| DHS 2014 (Namibia) [47] | cross-sectional study | 2013 | Yes | 2010-2015 | Men aged 15-49 | Namibia | urban and rural | 4021 |
| DHS 2016 (Lesotho) [48] | cross-sectional study | 2014 | Yes | 2010-2015 | Men aged 15-59 | Lesotho | urban and rural | 2,931 |
| DHS 2012 (Ethiopia) [49] | cross-sectional study | 2011 | Yes | 2010-2015 | Men aged 15-59 | Ethiopia | urban and rural | 14,110 |
| DHS 2017 (Ethiopia) [50] | cross-sectional study | 2016 | Yes | 2015-2023 | Men aged 15-59 | Ethiopia | urban and rural | 12,688 |
| DHS 2015 (Kenya) [51] | cross-sectional study | 2014 | Yes | 2010-2015 | Men aged 15-59 | Kenya | urban and rural | 12,819 |
| DHS 2012 (Rwanda) [52] | cross-sectional study | 2010 | Yes | 2010-2015 | Men aged 15-59 | Rwanda | Urban and rural | 6329 |
| DHS 2016 (Rwanda) [53] | cross-sectional study | 2014-2015 | Yes | 2010-2015 | Men aged 15-59 | Rwanda | Urban and rural | 6,217 |
| DHS 2021 (Rwanda) [54] | cross-sectional study | 2019-2020 | Yes | 2015-2023 | Men aged 15-59 | Rwanda | Urban and rural | 6513 |
| DHS 2019 (South Africa) [55] | cross-sectional study | 2016 | Yes | 2015-2023 | Men aged 15-59 | South Africa | Urban and rural | 3618 |
| DHS 2011 (Tanzania) [56] | cross-sectional study | 2010 | Yes | 2010-2015 | Men aged 15-49 | Tanzania | Urban and rural | 2527 |
| AIS 2013 (Tanzania) [57] | cross-sectional study | 2011-2012 | Yes | 2010-2015 | Men aged 15-49 | Tanzania | Urban and rural | 8352 |
| DHS 2016 (Tanzania) [58] | cross-sectional study | 2015-2016 | Yes | 2015-2023 | Men aged 15-49 | Tanzania | Urban and rural | 3514 |
| AIS 2012 (Uganda) [59] | cross-sectional study | 2011 | Yes | 2010-2015 | Men aged 15-59 | Uganda | Urban and rural | 2295 |
| DHS 2012 (Uganda) [60] | cross-sectional study | 2011 | Yes | 2010-2015 | Men aged 15-54 | Uganda | Urban and rural | 9588 |
| DHS 2018 (Uganda) [61] | cross-sectional study | 2016 | Yes | 2015-2023 | Men aged 15-54 | Uganda | Urban and rural | 5336 |
| DHS 2014 (Zambia) [62] | cross-sectional study | 2013-2014 | Yes | 2010-2015 | Men aged 15-59 | Zambia | Urban and rural | 14773 |
| DHS 2020 (Zambia) [63] | cross-sectional study | 2018 | Yes | 2015-2023 | Men aged 15-59 | Zambia | Urban and rural | 12132 |
| DHS 2012 (Zimbabwe) [64] | cross-sectional study | 2010-2011 | Yes | 2010-2015 | Men aged 15-54 | Zimbabwe | Urban and rural | 7480 |
| DHS 2016 (Zimbabwe) [65] | cross-sectional study | 2015 | Yes | 2015-2023 | Men aged 15-54 | Zimbabwe | Urban and rural | 8396 |
