# Supplementary material for: Estimates of the prevalence of male circumcision in sub-Saharan Africa from 2010–2023—A systematic review and meta-analysis
Source: PLoS One. 2024 Mar 13;19(3):e0298387. doi: 10.1371/journal.pone.0298387 (PMC10936832; doi:10.1371/journal.pone.0298387)
Supplement: S2 Table — This table shows the assessment of risk of bias of the included studies using 11 item Hoy et al tool. (DOCX) [file pone.0298387.s003.docx]

Supplementary Table 2 – Assessment of risk of bias of included studies using Hoy et al 2012

| Study | 1. was the study target population a close representation of the national population in relation to relevant variables? | 2. Was the sampling frame a true or close representation of the target population? | 3. Was some form of random selection used to select the sample, OR was a census undertaken? | 4. Was the likelihood of non-response bias minimal in the study? | 5. Were data collected directly from the subjects (as opposed to a proxy)? | 6. Was an acceptable case definition used in the study? | 7. Was the study instrument that measured the parameter of interest shown to have validity and reliability? | 8. Was the same mode of data collection used for all subjects? | 9. Was the length of the shortest prevalence period for the parameter of interest appropriate? | 10. Were the numerator(s) for the parameter of interest appropriate? | 11. Summary score on the overall risk of study bias | Risk of bias |
| --- | --- | --- | --- | --- | --- | --- | --- | --- | --- | --- | --- | --- |
| Auvert et al. 2013 [18] | 1 | 0 | 0 | 1 | 0 | 0 | 0 | 0 | 0 | 0 | 2 | Low risk |
| Kibira et al. 2014 [19] | 0 | 0 | 0 | 1 | 0 | 0 | 0 | 0 | 0 | 0 | 1 | Low risk |
| Ortblad et al. 2018 [20] | 1 | 0 | 0 | 0 | 0 | 1 | 0 | 0 | 0 | 0 | 2 | Low risk |
| Rupfutse et al. 2014 [21] | 1 | 1 | 0 | 1 | 0 | 1 | 1 | 0 | 0 | 0 | 5 | Moderate risk |
| Baisley et al. 2018 [22] | 1 | 0 | 0 | 0 | 0 | 1 | 0 | 0 | 0 | 0 | 2 | Low risk |
| Edossa et al. 2020 [23] | 1 | 0 | 0 | 1 | 0 | 0 | 0 | 0 | 0 | 0 | 2 | Low risk |
| Forbes et al. 2012 [24] | 1 | 0 | 0 | 0 | 0 | 0 | 0 | 0 | 0 | 0 | 1 | Low risk |
| Galbraith et al. 2014 [25] | 1 | 0 | 0 | 0 | 0 | 1 | 1 | 0 | 0 | 0 | 3 | Low risk |
| Hensen et al. 2019 [26] | 1 | 0 | 0 | 1 | 0 | 1 | 1 | 1 | 0 | 0 | 5 | Moderate risk |
| Kim et al. 2019 [27] | 0 | 0 | 0 | 1 | 0 | 0 | 1 | 0 | 0 | 0 | 2 | Low risk |
| Kong et al. 2014 [28] | 1 | 0 | 0 | 0 | 0 | 1 | 1 | 0 | 0 | 0 | 3 | Low risk |
| Marshall et al. 2017 [29] | 1 | 0 | 0 | 1 | 0 | 1 | 1 | 0 | 0 | 0 | 4 | Moderate risk |
| Mavhu et al. 2011 [30] | 1 | 1 | 0 | 0 | 0 | 1 | 1 | 0 | 0 | 0 | 4 | Moderate risk |
| Mutombo et al. 2015 [31] | 0 | 0 | 0 | 0 | 0 | 1 | 1 | 0 | 0 | 0 | 1 | Low risk |
| Nzamwita et al. 2021 [32] | 1 | 0 | 0 | 1 | 0 | 1 | 1 | 0 | 0 | 0 | 4 | Moderate risk |
| Odoyo-June et al. 2017 [33] | 1 | 0 | 0 | 1 | 0 | 0 | 0 | 0 | 0 | 0 | 2 | Low risk |
| Peltzer et al. 2014 [35] | 0 | 0 | 0 | 1 | 0 | 1 | 1 | 0 | 0 | 0 | 3 | Low risk |
| Tapera et al. 2017 [36] | 1 | 1 | 1 | 01 | 0 | 1 | 1 | 0 | 0 | 0 | 5 | Moderate risk |
| Westercamp et al. 2017 [37] | 1 | 1 | 0 | 1 | 0 | 0 | 0 | 0 | 0 | 0 | 3 | Low risk |
| Tram 2014 [38] | 0 | 0 | 0 | 1 | 0 | 1 | 1 | 1 | 0 | 0 | 4 | Moderate risk |
| Gasasira et al 2012 [39] | 0 | 0 | 0 | 1 | 0 | 1 | 1 | 1 | 0 | 0 | 4 | Moderate risk |
| Shezi et al 2023 [40] | 1 | 0 | 0 | 1 | 0 | 1 | 1 | 0 | 0 | 0 | 4 | Moderate risk |
| Wambura et al 2011 [16] | 1 | 1 | 0 | 1 | 0 | 1 | 1 | 0 | 0 | 0 | 5 | Moderate risk |
| Nanteza et al 2018 [41] | 1 | 0 | 1 | 1 | 0 | 1 | 1 | 0 | 0 | 0 | 5 | Moderate risk |
| Hatzold et al 2014 [42] | 0 | 1 | 0 | 1 | 0 | 0 | 1 | 0 | 0 | 0 | 3 | Low risk |
| Marukutira et al 2022 [43] | 1 | 0 | 0 | 1 | 0 | 0 | 1 | 0 | 0 | 0 | 3 | Low risk |
| Keetile, M 2020 [44] | 0 | 0 | 0 | 0 | 0 | 1 | 1 | 0 | 0 | 0 | 2 | Low risk |
| Zuma et al 2022 [17] | 0 | 0 | 0 | 0 | 0 | 1 | 1 | 0 | 0 | 0 | 2 | Low risk |
| Hines et al 2021 [45] | 0 | 0 | 0 | 0 | 0 | 1 | 1 | 0 | 0 | 0 | 2 | Low risk |
| DHS 2017 (Malawi) [46] | 0 | 0 | 0 | 0 | 0 | 1 | 1 | 0 | 0 | 0 | 2 | Low risk |
| DHS 2014 (Namibia) [47] | 0 | 0 | 0 | 0 | 0 | 1 | 1 | 0 | 0 | 0 | 2 | Low risk |
| DHS 2016 (Lesotho) [48] | 0 | 0 | 0 | 0 | 0 | 1 | 1 | 0 | 0 | 0 | 2 | Low risk |
| DHS 2012 (Ethiopia) [49] | 0 | 0 | 0 | 0 | 0 | 1 | 1 | 0 | 0 | 0 | 2 | Low risk |
| DHS 2017 (Ethiopia) [50] | 0 | 0 | 0 | 0 | 0 | 1 | 1 | 0 | 0 | 0 | 2 | Low risk |
| DHS 2015 (Kenya) [51] | 0 | 0 | 0 | 0 | 0 | 1 | 1 | 0 | 0 | 0 | 2 | Low risk |
| DHS 2012 (Rwanda) [52] | 0 | 0 | 0 | 0 | 0 | 1 | 1 | 0 | 0 | 0 | 2 | Low risk |
| DHS 2016 (Rwanda) [53] | 0 | 0 | 0 | 0 | 0 | 1 | 1 | 0 | 0 | 0 | 2 | Low risk |
| DHS 2021 (Rwanda) [54] | 0 | 0 | 0 | 0 | 0 | 1 | 1 | 0 | 0 | 0 | 2 | Low risk |
| DHS 2019 (South Africa) [55] | 0 | 0 | 0 | 0 | 0 | 1 | 1 | 0 | 0 | 0 | 2 | Low risk |
| DHS 2011 (Tanzania) [56] | 0 | 0 | 0 | 0 | 0 | 1 | 1 | 0 | 0 | 0 | 2 | Low risk |
| AIS 2013 (Tanzania) [57] | 0 | 0 | 0 | 0 | 0 | 1 | 1 | 0 | 0 | 0 | 2 | Low risk |
| DHS 2016 (Tanzania) [58] | 0 | 0 | 0 | 0 | 0 | 1 | 1 | 0 | 0 | 0 | 2 | Low risk |
| AIS 2012 (Uganda) [59] | 0 | 0 | 0 | 0 | 0 | 1 | 1 | 0 | 0 | 0 | 2 | Low risk |
| DHS 2012 (Uganda) [60] | 0 | 0 | 0 | 0 | 0 | 1 | 1 | 0 | 0 | 0 | 2 | Low risk |
| DHS 2018 (Uganda) [61] | 0 | 0 | 0 | 0 | 0 | 1 | 1 | 0 | 0 | 0 | 2 | Low risk |
| DHS 2014 (Zambia) [62] | 0 | 0 | 0 | 0 | 0 | 1 | 1 | 0 | 0 | 0 | 2 | Low risk |
| DHS 2020 (Zambia) [63] | 0 | 0 | 0 | 0 | 0 | 1 | 1 | 0 | 0 | 0 | 2 | Low risk |
| DHS 2012 (Zimbabwe) [64] | 0 | 0 | 0 | 0 | 0 | 1 | 1 | 0 | 0 | 0 | 2 | Low risk |
| DHS 2016 (Zimbabwe) [65] | 0 | 0 | 0 | 0 | 0 | 1 | 1 | 0 | 0 | 0 | 2 | Low risk |
