# Supplementary material for: Estimates of the prevalence of male circumcision in sub-Saharan Africa from 2010–2023—A systematic review and meta-analysis
Source: PLoS One. 2024 Mar 13;19(3):e0298387. doi: 10.1371/journal.pone.0298387 (PMC10936832; doi:10.1371/journal.pone.0298387)
Supplement: S3 Table — This table shows the prevalence of male circumcision in sub-Saharan African countries based on the rural and urban settings. (DOCX) [file pone.0298387.s004.docx]

Supplementary Table 3: Male circumcision prevalence in Urban vs Rural settings

| Setting | Percentage | L95%CI | U95%CI | %Weight |
| --- | --- | --- | --- | --- |
| Urban |  |  |  |  |
| Tram 2014 (Eswatini) | 13.3 | 11.482 | 15.356 | 0.44 |
| Tram 2014 (Namibia) | 25.9 | 25.297 | 26.512 | 7.51 |
| Tram 2014 (Zambia) | 13.1 | 11.858 | 14.451 | 0.98 |
| Keetile, M 2020 | 14.967 | 13.845 | 16.163 | 1.37 |
| DHS 2009 (Kenya) | 91.2 | 89.127 | 92.909 | 0.33 |
| DHS 2010 (Lesotho) | 33.8 | 30.69 | 37.056 | 0.32 |
| DHS 2010(Malawi) | 23.5 | 21.382 | 25.759 | 0.54 |
| DHS 2012 (Rwanda) | 31.6 | 28.8 | 34.54 | 0.38 |
| DHS 2011 (Tanzania) | 89.9 | 87.432 | 91.928 | 0.26 |
| DHS 2012 (Zimbabwe) | 9.7 | 8.625 | 10.893 | 0.99 |
| DHS 2012 (Ethiopia) | 92 | 91.108 | 92.809 | 1.47 |
| DHS 2011 (Mozambique) | 51.6 | 48.902 | 54.288 | 0.5 |
| DHS 2012 (Uganda) | 40.2 | 35.718 | 44.852 | 0.17 |
| Kibira et al. 2014 | 27.958 | 26.984 | 28.954 | 3 |
| Kim et al. 2019 | 73.503 | 72.546 | 74.439 | 3.14 |
| Keetile, M 2020 | 28.465 | 26.676 | 30.326 | 0.88 |
| DHS 2014 (Namibia) | 30 | 28.155 | 31.913 | 0.86 |
| DHS 2014 (Zambia) | 29.1 | 27.994 | 30.232 | 2.38 |
| DHS 2015 (Kenya) | 93.7 | 93.014 | 94.323 | 2 |
| DHS 2016 (Lesotho) | 68.4 | 65.325 | 71.322 | 0.35 |
| DHS 2016 (Rwanda) | 58.2 | 55.35 | 60.996 | 0.44 |
| DHS 2016 (Zimbabwe) | 18 | 16.644 | 19.44 | 1.09 |
| DHS 2017 (Malawi) | 36 | 33.473 | 38.607 | 0.5 |
| Kim et al. 2019 (B) | 79.994 | 78.639 | 81.284 | 1.32 |
| DHS 2016 (Tanzania) | 91.3 | 89.609 | 92.738 | 0.47 |
| DHS 2017 (Ethiopia) | 96.3 | 95.449 | 96.997 | 0.87 |
| DHS 2019 (South Africa) | 57.1 | 55.023 | 59.153 | 0.83 |
| DHS 2018 (Uganda) | 55.7 | 52.959 | 58.406 | 0.48 |
| DHS 2020 (Zambia) | 39.9 | 38.553 | 41.263 | 1.89 |
| DHS 2021 (Rwanda) | 74.6 | 71.963 | 77.068 | 0.42 |
| Subgroup, IVhet | 45.256 | 27.674 | 63.433 | 36.18 |
| Subgroup, DL | 50.541 | 38.891 | 62.162 |  |
| Rural |  |  |  |  |
| Tram 2014 (Eswatini) | 6.2 | 5.389 | 7.124 | 1.12 |
| Tram 2014 (Namibia) | 16.1 | 14.537 | 17.796 | 0.74 |
| Tram 2014 (Zambia) | 12.4 | 11.334 | 13.551 | 1.28 |
| Keetile, M 2020 | 9.694 | 8.59 | 10.922 | 0.93 |
| DHS 2009 (Kenya) | 83.8 | 82.269 | 85.222 | 0.9 |
| DHS 2010 (Lesotho) | 58.6 | 56.51 | 60.659 | 0.81 |
| DHS 2010(Malawi) | 20.9 | 19.834 | 22.007 | 2.03 |
| DHS 2012 (Rwanda) | 9.9 | 9.126 | 10.731 | 2 |
| DHS 2011 (Tanzania) | 65.6 | 63.395 | 67.739 | 0.69 |
| DHS 2012 (Zimbabwe) | 8.7 | 7.91 | 9.56 | 1.69 |
| DHS 2012 (Ethiopia) | 92 | 91.42 | 92.544 | 3.37 |
| DHS 2011 (Mozambique) | 44.8 | 42.73 | 46.889 | 0.83 |
| DHS 2012 (Uganda) | 23.4 | 21.467 | 25.45 | 0.65 |
| Keetile, M 2020 | 26.813 | 24.589 | 29.159 | 0.54 |
| DHS 2014 (Namibia) | 19.7 | 17.898 | 21.635 | 0.65 |
| DHS 2014 (Zambia) | 15.7 | 14.88 | 16.556 | 2.72 |
| DHS 2015 (Kenya) | 91.7 | 91.019 | 92.334 | 2.55 |
| DHS 2016 (Lesotho) | 74.3 | 72.195 | 76.298 | 0.66 |
| DHS 2016 (Rwanda) | 22 | 20.802 | 23.247 | 1.66 |
| DHS 2016 (Zimbabwe) | 12.2 | 11.333 | 13.123 | 1.94 |
| DHS 2017 (Malawi) | 25.9 | 24.788 | 27.044 | 2.18 |
| DHS 2016 (Tanzania) | 74.2 | 72.357 | 75.961 | 0.85 |
| DHS 2017 (Ethiopia) | 90 | 89.374 | 90.593 | 3.5 |
| DHS 2019 (South Africa) | 56.7 | 53.608 | 59.741 | 0.38 |
| DHS 2018 (Uganda) | 42.5 | 40.929 | 44.086 | 1.42 |
| DHS 2020 (Zambia) | 25.2 | 24.132 | 26.299 | 2.32 |
| DHS 2021 (Rwanda) | 51.6 | 50.175 | 53.022 | 1.78 |
| Subgroup, IVhet | 42.597 | 26.532 | 59.461 | 40.19 |
| Subgroup, DL | 38.788 | 25.209 | 53.277 |  |
| Urban and rural |  |  |  |  |
| Peltzer et al. 2014 | 42.801 | 41.617 | 43.994 | 2.51 |
| Mutombo et al. 2015 | 19.068 | 17.929 | 20.262 | 1.64 |
| Gasasira et al 2012 | 17.031 | 14.923 | 19.368 | 0.41 |
| Hatzold et al 2014 | 11.33 | 9.636 | 13.279 | 0.44 |
| Marukutira et al 2022 | 50.124 | 49.26 | 50.988 | 4.84 |
| zuma et al 2022 | 61.6 | 61.101 | 62.097 | 13.79 |
| Subgroup, IVhet | 52.456 | 28.332 | 76.042 | 23.63 |
| Subgroup, DL | 31.876 | 18.391 | 47.024 |  |
| Overall, IVhet | 45.92 | 33.527 | 58.567 | 100 |
| Overall, DL | 43.757 | 36.057 | 51.61 |  |
