# Supplementary material for: Estimates of the prevalence of male circumcision in sub-Saharan Africa from 2010–2023—A systematic review and meta-analysis
Source: PLoS One. 2024 Mar 13;19(3):e0298387. doi: 10.1371/journal.pone.0298387 (PMC10936832; doi:10.1371/journal.pone.0298387)
Supplement: S4 Table — This table shows the prevalence of male circumcision in Sub-Saharan African countries based on the region. (DOCX) [file pone.0298387.s005.docx]

Supplementary Table 4: Male circumcision prevalence in Eastern Vs Southern regions

| Region and Study | Percentage | L95%CI | U95%CI | % Weight |
| --- | --- | --- | --- | --- |
| South |  |  |  |  |
| Tram 2014 (Eswatini) | 8.229 | 7.432 | 9.104 | 1.57 |
| Tram 2014 (Namibia) | 25.029 | 24.459 | 25.606 | 8.3 |
| Tram 2014 (Zambia) | 12.708 | 11.889 | 13.575 | 2.27 |
| Keetile, M 2020 | 12.48 | 11.686 | 13.32 | 2.38 |
| Peltzer et al. 2014 | 42.801 | 41.617 | 43.994 | 2.52 |
| DHS 2010 (Lesotho) | 51.646 | 49.859 | 53.429 | 1.14 |
| Mutombo et al. 2015 | 19.068 | 17.929 | 20.262 | 1.65 |
| DHS 2010(Malawi) | 21.455 | 20.497 | 22.445 | 2.58 |
| DHS 2012 (Zimbabwe) | 9.073 | 8.427 | 9.763 | 2.69 |
| DHS 2011 (Mozambique) | 52.66 | 50.377 | 54.932 | 0.7 |
| Keetile, M 2020 | 24.954 | 23.601 | 26.357 | 1.43 |
| DHS 2014 (Namibia) | 25.541 | 24.217 | 26.912 | 1.52 |
| Hatzold et al 2014 | 11.33 | 9.636 | 13.279 | 0.44 |
| DHS 2014 (Zambia) | 21.953 | 21.264 | 22.657 | 5.13 |
| Marukutira et al 2022 | 50.124 | 49.26 | 50.988 | 4.87 |
| DHS 2016 (Lesotho) | 72.266 | 70.534 | 73.934 | 1.01 |
| DHS 2016 (Zimbabwe) | 14.291 | 13.543 | 15.073 | 3.04 |
| DHS 2017 (Malawi) | 27.792 | 26.764 | 28.844 | 2.7 |
| DHS 2019 (South Africa) | 56.964 | 55.242 | 58.67 | 1.21 |
| zuma et al 2022 | 61.6 | 61.101 | 62.097 | 13.87 |
| DHS 2020 (Zambia) | 31.795 | 30.938 | 32.664 | 4.23 |
|  |  |  |  |  |
| Subgroup, IVhet | 33.41 | 21.705 | 46.212 | 65.27 |
| Subgroup, DL | 29.479 | 21.32 | 38.344 |  |
|  |  |  |  |  |
| East |  |  |  |  |
| DHS 2009 (Kenya) | 85.758 | 84.516 | 86.916 | 1.23 |
| Gasasira et al 2012 | 17.031 | 14.923 | 19.368 | 0.42 |
| DHS 2012 (Rwanda) | 13.351 | 12.535 | 14.211 | 2.4 |
| DHS 2011 (Tanzania) | 72.26 | 70.481 | 73.97 | 0.96 |
| DHS 2012 (Ethiopia) | 92.003 | 91.522 | 92.46 | 4.87 |
| DHS 2012 (Uganda) | 26.783 | 24.964 | 28.685 | 0.82 |
| Kibira et al. 2014 | 27.958 | 26.984 | 28.954 | 3.02 |
| Kim et al. 2019 | 73.503 | 72.546 | 74.439 | 3.16 |
| DHS 2015 (Kenya) | 92.58 | 92.099 | 93.034 | 4.57 |
| DHS 2016 (Rwanda) | 29.586 | 28.402 | 30.797 | 2.11 |
| Kim et al. 2019 (B) | 79.994 | 78.639 | 81.284 | 1.33 |
| DHS 2016 (Tanzania) | 80.279 | 78.931 | 81.561 | 1.33 |
| DHS 2017 (Ethiopia) | 91.254 | 90.726 | 91.754 | 4.39 |
| DHS 2018 (Uganda) | 45.841 | 44.468 | 47.219 | 1.91 |
| DHS 2021 (Rwanda) | 55.987 | 54.711 | 57.255 | 2.21 |
|  |  |  |  |  |
| Subgroup, IVhet | 69.899 | 49.861 | 86.841 | 34.73 |
| Subgroup, DL | 60.78 | 43.354 | 76.965 |  |
|  |  |  |  |  |
| Overall, IVhet | 45.903 | 32.272 | 59.841 | 100 |
| Overall, DL | 42.123 | 32.126 | 52.449 |  |
