# Supplementary material for: Estimates of the prevalence of male circumcision in sub-Saharan Africa from 2010–2023—A systematic review and meta-analysis
Source: PLoS One. 2024 Mar 13;19(3):e0298387. doi: 10.1371/journal.pone.0298387 (PMC10936832; doi:10.1371/journal.pone.0298387)
Supplement: S5 Table — This table shows the prevalence of male circumcision in Sub-Saharan Africa among different age groups. (DOCX) [file pone.0298387.s006.docx]

Supplementary Table 5: Male circumcision prevalence in different age groups

| Age group and Study | Percentage | L95%CI | U95%CI | % Weight |
| --- | --- | --- | --- | --- |
| 15-19 |  |  |  |  |
| Tram 2014 (Eswatini) | 4.2 | 3.245 | 5.42 | 0.51 |
| Tram 2014 (Namibia) | 16.6 | 14.324 | 19.157 | 0.35 |
| Tram 2014 (Zambia) | 10.1 | 8.637 | 11.779 | 0.55 |
| DHS 2009 (Kenya) | 75.5 | 72.353 | 78.396 | 0.3 |
| DHS 2010 (Lesotho) | 26.8 | 23.907 | 29.905 | 0.32 |
| DHS 2010(Malawi) | 21.7 | 19.831 | 23.693 | 0.67 |
| DHS 2012 (Rwanda) | 10 | 8.559 | 11.653 | 0.56 |
| DHS 2011 (Tanzania) | 64.3 | 60.528 | 67.903 | 0.25 |
| DHS 2012 (Zimbabwe) | 5.3 | 4.341 | 6.456 | 0.67 |
| DHS 2012 (Ethiopia) | 92 | 90.943 | 92.943 | 1.09 |
| DHS 2011 (Mozambique) | 36.2 | 33.098 | 39.421 | 0.34 |
| DHS 2012 (Uganda) | 27.3 | 23.756 | 31.157 | 0.21 |
| DHS 2014 (Namibia) | 21 | 19.146 | 22.983 | 0.67 |
| DHS 2014 (Zambia) | 22.7 | 21.311 | 24.152 | 1.29 |
| DHS 2015 (Kenya) | 87.1 | 85.74 | 88.348 | 0.98 |
| DHS 2016 (Lesotho) | 58.9 | 55.192 | 62.509 | 0.27 |
| DHS 2016 (Rwanda) | 26.5 | 24.157 | 28.983 | 0.49 |
| DHS 2016 (Zimbabwe) | 22.6 | 20.873 | 24.426 | 0.82 |
| DHS 2017 (Malawi) | 28.6 | 26.57 | 30.721 | 0.7 |
| DHS 2016 (Tanzania) | 80.2 | 77.52 | 82.632 | 0.36 |
| DHS 2017 (Ethiopia) | 86.3 | 84.917 | 87.575 | 0.99 |
| DHS 2019 (South Africa) | 57.4 | 53.557 | 61.156 | 0.25 |
| DHS 2018 (Uganda) | 45.5 | 42.798 | 48.229 | 0.5 |
| DHS 2020 (Zambia) | 35.9 | 34.138 | 37.701 | 1.07 |
| DHS 2021 (Rwanda) | 72.6 | 70.308 | 74.779 | 0.59 |
| Subgroup, IVhet | 41.874 | 27.101 | 57.395 | 14.81 |
| Subgroup, DL | 40.186 | 27.105 | 53.991 |  |
| 20-24 |  |  |  |  |
| Tram 2014 (Eswatini) | 6.5 | 5.057 | 8.318 | 0.34 |
| Tram 2014 (Namibia) | 21.9 | 19.087 | 24.999 | 0.29 |
| Tram 2014 (Zambia) | 13.2 | 12.553 | 13.875 | 3.88 |
| DHS 2009 (Kenya) | 89.4 | 86.753 | 91.57 | 0.24 |
| DHS 2010 (Lesotho) | 62.303 | 58.467 | 65.99 | 0.24 |
| DHS 2010(Malawi) | 22.2 | 19.974 | 24.598 | 0.48 |
| DHS 2012 (Rwanda) | 16.1 | 14.095 | 18.329 | 0.45 |
| DHS 2011 (Tanzania) | 74.5 | 70.09 | 78.46 | 0.16 |
| DHS 2012 (Zimbabwe) | 8.1 | 6.771 | 9.663 | 0.53 |
| DHS 2012 (Ethiopia) | 92 | 90.828 | 93.034 | 0.9 |
| DHS 2011 (Mozambique) | 44 | 40.187 | 47.885 | 0.25 |
| DHS 2012 (Uganda) | 29.8 | 25.038 | 35.044 | 0.12 |
| DHS 2014 (Namibia) | 22.8 | 20.208 | 25.618 | 0.36 |
| DHS 2014 (Zambia) | 27.6 | 25.825 | 29.449 | 0.9 |
| DHS 2015 (Kenya) | 96.5 | 95.631 | 97.201 | 0.82 |
| DHS 2016 (Lesotho) | 83 | 79.67 | 85.881 | 0.22 |
| DHS 2016 (Rwanda) | 44.2 | 41.141 | 47.304 | 0.38 |
| DHS 2016 (Zimbabwe) | 12.8 | 11.111 | 14.703 | 0.51 |
| DHS 2017 (Malawi) | 29.5 | 27.176 | 31.935 | 0.54 |
| DHS 2016 (Tanzania) | 84.2 | 80.996 | 86.951 | 0.22 |
| DHS 2017 (Ethiopia) | 90.1 | 88.668 | 91.368 | 0.73 |
| DHS 2019 (South Africa) | 62.5 | 58.518 | 66.32 | 0.23 |
| DHS 2018 (Uganda) | 53.2 | 50.019 | 56.355 | 0.37 |
| DHS 2020 (Zambia) | 39.4 | 37.297 | 41.543 | 0.78 |
| DHS 2021 (Rwanda) | 74.5 | 71.649 | 77.156 | 0.37 |
| Subgroup, IVhet | 39.161 | 16.464 | 64.351 | 14.32 |
| Subgroup, DL | 48.188 | 33.09 | 63.45 |  |
| 25-29 |  |  |  |  |
| Tram 2014 (Eswatini) | 7.9 | 6.032 | 10.283 | 0.24 |
| Tram 2014 (Namibia) | 21.7 | 18.809 | 24.899 | 0.27 |
| Tram 2014 (Zambia) | 12.1 | 10.202 | 14.295 | 0.38 |
| DHS 2009 (Kenya) | 85 | 81.54 | 87.908 | 0.19 |
| DHS 2010 (Lesotho) | 61.7 | 57.193 | 66.014 | 0.18 |
| DHS 2010(Malawi) | 18.3 | 16.126 | 20.695 | 0.42 |
| DHS 2012 (Rwanda) | 17.2 | 15.025 | 19.617 | 0.4 |
| DHS 2011 (Tanzania) | 75.4 | 70.577 | 79.66 | 0.13 |
| DHS 2012 (Zimbabwe) | 10.6 | 9.004 | 12.44 | 0.48 |
| DHS 2012 (Ethiopia) | 92 | 90.813 | 93.046 | 0.88 |
| DHS 2011 (Mozambique) | 52.6 | 48.412 | 56.752 | 0.21 |
| DHS 2012 (Uganda) | 27.7 | 23.337 | 32.533 | 0.14 |
| DHS 2014 (Namibia) | 27.8 | 24.513 | 31.344 | 0.25 |
| DHS 2014 (Zambia) | 22.6 | 20.796 | 24.512 | 0.75 |
| DHS 2015 (Kenya) | 94.6 | 93.55 | 95.487 | 0.81 |
| DHS 2016 (Lesotho) | 76.5 | 72.162 | 80.346 | 0.16 |
| DHS 2016 (Rwanda) | 35.3 | 32.32 | 38.399 | 0.37 |
| DHS 2016 (Zimbabwe) | 10 | 8.396 | 11.871 | 0.44 |
| DHS 2017 (Malawi) | 30.2 | 27.464 | 33.085 | 0.39 |
| DHS 2016 (Tanzania) | 80.1 | 76.304 | 83.42 | 0.19 |
| DHS 2017 (Ethiopia) | 91.5 | 90.189 | 92.65 | 0.76 |
| DHS 2019 (South Africa) | 62.8 | 58.507 | 66.9 | 0.2 |
| DHS 2018 (Uganda) | 49.2 | 45.614 | 52.794 | 0.29 |
| DHS 2020 (Zambia) | 31.5 | 29.349 | 33.734 | 0.66 |
| DHS 2021 (Rwanda) | 61 | 57.362 | 64.519 | 0.27 |
| Subgroup, IVhet | 48.984 | 31.542 | 66.547 | 9.46 |
| Subgroup, DL | 46.024 | 31.168 | 61.235 |  |
| 30-34 |  |  |  |  |
| Tram 2014 (Eswatini) | 9.9 | 7.425 | 13.084 | 0.17 |
| DHS 2012 (Rwanda) | 17.7 | 15.069 | 20.679 | 0.27 |
| DHS 2011 (Tanzania) | 75 | 70.223 | 79.237 | 0.14 |
| DHS 2016 (Rwanda) | 27.7 | 24.92 | 30.663 | 0.36 |
| Subgroup, IVhet | 25.577 | 4.489 | 54.13 | 0.94 |
| Subgroup, DL | 29.593 | 8.162 | 56.417 |  |
| 35-39 |  |  |  |  |
| Tram 2014 (Eswatini) | 19.7 | 15.954 | 24.074 | 0.14 |
| DHS 2012 (Rwanda) | 12.8 | 10.138 | 16.036 | 0.19 |
| DHS 2011 (Tanzania) | 72.1 | 66.77 | 76.871 | 0.12 |
| DHS 2016 (Rwanda) | 22 | 18.785 | 25.592 | 0.22 |
| Subgroup, IVhet | 24.747 | 4.819 | 51.687 | 0.67 |
| Subgroup, DL | 28.938 | 7.885 | 55.473 |  |
| 40-44 |  |  |  |  |
| Tram 2014 (Eswatini) | 12.5 | 9.068 | 16.988 | 0.1 |
| DHS 2012 (Rwanda) | 12.4 | 9.611 | 15.856 | 0.17 |
| DHS 2011 (Tanzania) | 74.7 | 69.193 | 79.514 | 0.1 |
| DHS 2016 (Rwanda) | 18.9 | 15.628 | 22.673 | 0.18 |
| Subgroup, IVhet | 22.323 | 1.453 | 53.698 | 0.56 |
| Subgroup, DL | 25.82 | 3.54 | 56.519 |  |
| 45-49 |  |  |  |  |
| Tram 2014 (Eswatini) | 11.9 | 8.486 | 16.44 | 0.1 |
| DHS 2012 (Rwanda) | 10 | 7.463 | 13.276 | 0.16 |
| DHS 2011 (Tanzania) | 79.8 | 73.763 | 84.735 | 0.08 |
| DHS 2016 (Rwanda) | 17.5 | 14.031 | 21.611 | 0.15 |
| Subgroup, IVhet | 19.285 | 0.009 | 53.263 | 0.49 |
| Subgroup, DL | 25.398 | 2.067 | 58.849 |  |
| 30-39 |  |  |  |  |
| Tram 2014 (Namibia) | 23.2 | 20.672 | 25.936 | 0.38 |
| Tram 2014 (Zambia) | 14.1 | 12.514 | 15.851 | 0.64 |
| DHS 2009 (Kenya) | 89.2 | 86.868 | 91.16 | 0.31 |
| DHS 2010 (Lesotho) | 59.475 | 55.759 | 63.086 | 0.26 |
| DHS 2010(Malawi) | 22.5 | 20.603 | 24.518 | 0.67 |
| DHS 2012 (Zimbabwe) | 11.2 | 9.824 | 12.741 | 0.69 |
| DHS 2012 (Ethiopia) | 92 | 91.019 | 92.882 | 1.26 |
| DHS 2011 (Mozambique) | 51.8 | 48.576 | 55.009 | 0.36 |
| DHS 2012 (Uganda) | 25.6 | 22.25 | 29.265 | 0.23 |
| DHS 2014 (Namibia) | 27.4 | 24.684 | 30.295 | 0.37 |
| DHS 2014 (Zambia) | 20.2 | 18.919 | 21.545 | 1.39 |
| DHS 2015 (Kenya) | 93.4 | 92.497 | 94.201 | 1.26 |
| DHS 2016 (Lesotho) | 75 | 71.415 | 78.273 | 0.24 |
| DHS 2016 (Zimbabwe) | 11.6 | 10.281 | 13.064 | 0.79 |
| DHS 2017 (Malawi) | 26.2 | 24.225 | 28.276 | 0.7 |
| DHS 2016 (Tanzania) | 82 | 79.318 | 84.403 | 0.34 |
| DHS 2017 (Ethiopia) | 93.9 | 92.989 | 94.699 | 1.17 |
| DHS 2019 (South Africa) | 51.8 | 48.43 | 55.153 | 0.33 |
| DHS 2018 (Uganda) | 43.1 | 40.354 | 45.889 | 0.47 |
| DHS 2020 (Zambia) | 28.4 | 26.719 | 30.143 | 1.03 |
| DHS 2021 (Rwanda) | 43.9 | 41.507 | 46.322 | 0.63 |
| Subgroup, IVhet | 49.963 | 30.845 | 69.086 | 13.51 |
| Subgroup, DL | 47.473 | 31.036 | 64.183 |  |
| 40-49 |  |  |  |  |
| Tram 2014 (Namibia) | 22.2 | 18.973 | 25.801 | 0.22 |
| Tram 2014 (Zambia) | 14.3 | 12.125 | 16.791 | 0.33 |
| DHS 2009 (Kenya) | 91.6 | 89.017 | 93.619 | 0.22 |
| DHS 2010 (Lesotho) | 62 | 57.081 | 66.684 | 0.15 |
| DHS 2010(Malawi) | 21.7 | 19.24 | 24.38 | 0.38 |
| DHS 2012 (Zimbabwe) | 11.5 | 9.641 | 13.664 | 0.37 |
| DHS 2012 (Ethiopia) | 92 | 90.783 | 93.068 | 0.84 |
| DHS 2011 (Mozambique) | 56.8 | 52.516 | 60.984 | 0.2 |
| DHS 2012 (Uganda) | 24.4 | 20.183 | 29.176 | 0.13 |
| DHS 2014 (Namibia) | 30.3 | 26.929 | 33.898 | 0.26 |
| DHS 2014 (Zambia) | 17.4 | 15.922 | 18.984 | 0.91 |
| DHS 2015 (Kenya) | 91.9 | 90.63 | 93.011 | 0.78 |
| DHS 2016 (Lesotho) | 71.9 | 67.236 | 76.136 | 0.15 |
| DHS 2016 (Zimbabwe) | 10.4 | 8.908 | 12.109 | 0.54 |
| DHS 2017 (Malawi) | 24.9 | 22.405 | 27.574 | 0.41 |
| DHS 2016 (Tanzania) | 73.8 | 70.281 | 77.038 | 0.25 |
| DHS 2017 (Ethiopia) | 94.4 | 93.348 | 95.294 | 0.83 |
| DHS 2019 (South Africa) | 53.6 | 49.652 | 57.504 | 0.24 |
| DHS 2018 (Uganda) | 39.4 | 36.136 | 42.762 | 0.32 |
| DHS 2020 (Zambia) | 22.8 | 21.006 | 24.699 | 0.76 |
| DHS 2021 (Rwanda) | 29.5 | 26.785 | 32.369 | 0.39 |
| Subgroup, IVhet | 49.087 | 29.219 | 69.095 | 8.7 |
| Subgroup, DL | 45.835 | 29.095 | 63.046 |  |
| 50-59 |  |  |  |  |
| Tram 2014 (Zambia) | 14 | 11.245 | 17.299 | 0.2 |
| Subgroup, IVhet | 14 | 11.245 | 17.299 | 0.2 |
| Subgroup, DL | 14 | 11.245 | 17.299 |  |
| 25 -34 |  |  |  |  |
| Keetile, M 2020 | 15.602 | 13.906 | 17.462 | 0.62 |
| Keetile, M 2020 | 27.6 | 24.858 | 30.522 | 0.37 |
| Subgroup, IVhet | 19.131 | 8.571 | 32.404 | 0.99 |
| Subgroup, DL | 20.673 | 10.011 | 33.763 |  |
| 35 -44 |  |  |  |  |
| Keetile, M 2020 | 18.322 | 15.973 | 20.931 | 0.36 |
| Keetile, M 2020 | 29.742 | 26.428 | 33.284 | 0.26 |
| Subgroup, IVhet | 22.44 | 12.148 | 34.681 | 0.62 |
| Subgroup, DL | 23.329 | 12.969 | 35.533 |  |
| 45 - 54 |  |  |  |  |
| Keetile, M 2020 | 17.549 | 14.683 | 20.837 | 0.23 |
| Keetile, M 2020 | 30.365 | 26.058 | 35.047 | 0.15 |
| Subgroup, IVhet | 21.862 | 10.467 | 35.799 | 0.38 |
| Subgroup, DL | 23.06 | 11.556 | 36.92 |  |
| 55 - 64 |  |  |  |  |
| Keetile, M 2020 | 20.489 | 16.418 | 25.265 | 0.12 |
| Keetile, M 2020 | 35.414 | 29.892 | 41.354 | 0.1 |
| Subgroup, IVhet | 26.39 | 12.933 | 42.385 | 0.22 |
| Subgroup, DL | 27.059 | 13.504 | 43.062 |  |
| 15-49 |  |  |  |  |
| Peltzer et al. 2014 | 42.801 | 41.617 | 43.994 | 2.57 |
| Kim et al. 2019 | 73.503 | 72.546 | 74.439 | 3.22 |
| Hatzold et al 2014 | 11.33 | 9.636 | 13.279 | 0.45 |
| Kim et al. 2019 (B) | 79.994 | 78.639 | 81.284 | 1.36 |
| Subgroup, IVhet | 61.903 | 30.843 | 88.91 | 7.6 |
| Subgroup, DL | 51.424 | 25.392 | 77.092 |  |
| 50-54 |  |  |  |  |
| DHS 2009 (Kenya) | 88.2 | 83.094 | 91.914 | 0.08 |
| DHS 2010(Malawi) | 24.2 | 20.047 | 28.902 | 0.14 |
| DHS 2012 (Rwanda) | 8.9 | 6.441 | 12.175 | 0.15 |
| Subgroup, IVhet | 30.374 | 0 | 84.764 | 0.37 |
| Subgroup, DL | 39.399 | 0 | 91.606 |  |
| 15+ |  |  |  |  |
| Mutombo et al. 2015 | 19.068 | 17.929 | 20.262 | 1.68 |
| Subgroup, IVhet | 19.068 | 17.929 | 20.262 | 1.68 |
| Subgroup, DL | 19.068 | 17.929 | 20.262 |  |
| all |  |  |  |  |
| Gasasira et al 2012 | 17.031 | 14.923 | 19.368 | 0.42 |
| zuma et al 2022 | 61.6 | 61.101 | 62.097 | 14.13 |
| Subgroup, IVhet | 60.246 | 0 | 100 | 14.56 |
| Subgroup, DL | 38.014 | 0 | 88.012 |  |
| 55-59 |  |  |  |  |
| DHS 2012 (Rwanda) | 6 | 3.697 | 9.594 | 0.1 |
| Subgroup, IVhet | 6 | 3.697 | 9.594 | 0.1 |
| Subgroup, DL | 6 | 3.697 | 9.594 |  |
| 50 -64 |  |  |  |  |
| DHS 2011 (Mozambique) | 55.3 | 51.016 | 59.507 | 0.2 |
| Subgroup, IVhet | 55.3 | 51.016 | 59.507 | 0.2 |
| Subgroup, DL | 55.3 | 51.016 | 59.507 |  |
| 15-59 |  |  |  |  |
| Kibira et al. 2014 | 27.958 | 26.984 | 28.954 | 3.07 |
| Subgroup, IVhet | 27.958 | 26.984 | 28.954 | 3.07 |
| Subgroup, DL | 27.958 | 26.984 | 28.954 |  |
| 16-49 |  |  |  |  |
| Marukutira et al 2022 | 50.124 | 49.26 | 50.988 | 4.96 |
| Subgroup, IVhet | 50.124 | 49.26 | 50.988 | 4.96 |
| Subgroup, DL | 50.124 | 49.26 | 50.988 |  |
