# Supplementary material for: Estimates of the prevalence of male circumcision in sub-Saharan Africa from 2010–2023—A systematic review and meta-analysis
Source: PLoS One. 2024 Mar 13;19(3):e0298387. doi: 10.1371/journal.pone.0298387 (PMC10936832; doi:10.1371/journal.pone.0298387)
Supplement: S7 Table — This table shows the prevalence of male circumcision in Sub-Saharan African countries by the time period: before 2015 and after 2015. (DOCX) [file pone.0298387.s008.docx]

Supplementary Table 7: Male circumcision prevalence by time period

| Study | Percentage | [95% Conf. | Interval] | Ivhet % weight |
| --- | --- | --- | --- | --- |
| Before 2015 |  |  |  |  |
| Tram 2014 (Eswatini) | 8.218 | 7.421 | 9.092 | 1.57 |
| Tram 2014 (Namibia) | 25.027 | 24.458 | 25.604 | 8.3 |
| Tram 2014 (Zambia) | 12.704 | 11.885 | 13.571 | 2.27 |
| Keetile, M 2020 | 12.48 | 11.686 | 13.32 | 2.38 |
| Peltzer et al. 2014 | 42.801 | 41.617 | 43.994 | 2.52 |
| DHS 2009 (Kenya) | 85.767 | 84.525 | 86.925 | 1.23 |
| DHS 2010 (Lesotho) | 51.631 | 49.844 | 53.414 | 1.14 |
| Mutombo et al. 2015 | 19.068 | 17.929 | 20.262 | 1.65 |
| DHS 2010(Malawi) | 21.449 | 20.491 | 22.439 | 2.58 |
| Gasasira et al 2012 | 17.031 | 14.923 | 19.368 | 0.42 |
| DHS 2012 (Rwanda) | 13.346 | 12.53 | 14.206 | 2.4 |
| DHS 2011 (Tanzania) | 72.264 | 70.486 | 73.975 | 0.96 |
| DHS 2012 (Zimbabwe) | 9.069 | 8.423 | 9.759 | 2.69 |
| DHS 2012 (Ethiopia) | 92 | 91.519 | 92.456 | 4.87 |
| DHS 2011 (Mozambique) | 52.651 | 50.367 | 54.923 | 0.7 |
| DHS 2012 (Uganda) | 26.794 | 24.974 | 28.696 | 0.82 |
| Kibira et al. 2014 | 27.958 | 26.984 | 28.954 | 3.02 |
| Kim et al. 2019 | 73.503 | 72.546 | 74.439 | 3.16 |
| Keetile, M 2020 | 24.954 | 23.601 | 26.357 | 1.43 |
| DHS 2014 (Namibia) | 25.545 | 24.221 | 26.916 | 1.52 |
| Hatzold et al 2014 | 11.33 | 9.636 | 13.279 | 0.44 |
| DHS 2014 (Zambia) | 21.951 | 21.262 | 22.655 | 5.13 |
| Marukutira et al 2022 | 50.124 | 49.26 | 50.988 | 4.87 |
| DHS 2015 (Kenya) | 92.579 | 92.097 | 93.033 | 4.57 |
| DHS 2016 (Lesotho) | 72.26 | 70.528 | 73.928 | 1.01 |
| DHS 2016 (Rwanda) | 29.588 | 28.404 | 30.8 | 2.11 |
| Subgroup, IVhet | 40.156 | 24.993 | 56.297 | 63.77 |
| Subgroup, DL | 37.078 | 25.073 | 49.924 |  |
| After 2015 |  |  |  |  |
| DHS 2016 (Zimbabwe) | 14.292 | 13.544 | 15.074 | 3.04 |
| DHS 2017 (Malawi) | 27.799 | 26.771 | 28.85 | 2.7 |
| Kim et al. 2019 (B) | 79.994 | 78.639 | 81.284 | 1.33 |
| DHS 2016 (Tanzania) | 80.288 | 78.94 | 81.57 | 1.33 |
| DHS 2017 (Ethiopia) | 91.25 | 90.722 | 91.751 | 4.39 |
| DHS 2019 (South Africa) | 56.975 | 55.253 | 58.681 | 1.21 |
| DHS 2018 (Uganda) | 45.839 | 44.466 | 47.217 | 1.91 |
| Zuma et al 2022 | 61.6 | 61.101 | 62.097 | 13.87 |
| DHS 2020 (Zambia) | 31.793 | 30.936 | 32.662 | 4.23 |
| DHS 2021 (Rwanda) | 55.987 | 54.711 | 57.255 | 2.21 |
| Subgroup, IVhet | 56.212 | 31.484 | 79.544 | 36.23 |
| Subgroup, DL | 55.374 | 38.08 | 72.057 |  |
| Overall, IVhet | 45.901 | 32.27 | 59.84 | 100 |
| Overall, DL | 42.122 | 32.125 | 52.448 |  |
