# Supplementary figures and images for: Estimates of the prevalence of male circumcision in sub-Saharan Africa from 2010–2023—A systematic review and meta-analysis
Source: PLoS One. 2024 Mar 13;19(3):e0298387. doi: 10.1371/journal.pone.0298387 (PMC10936832; doi:10.1371/journal.pone.0298387)

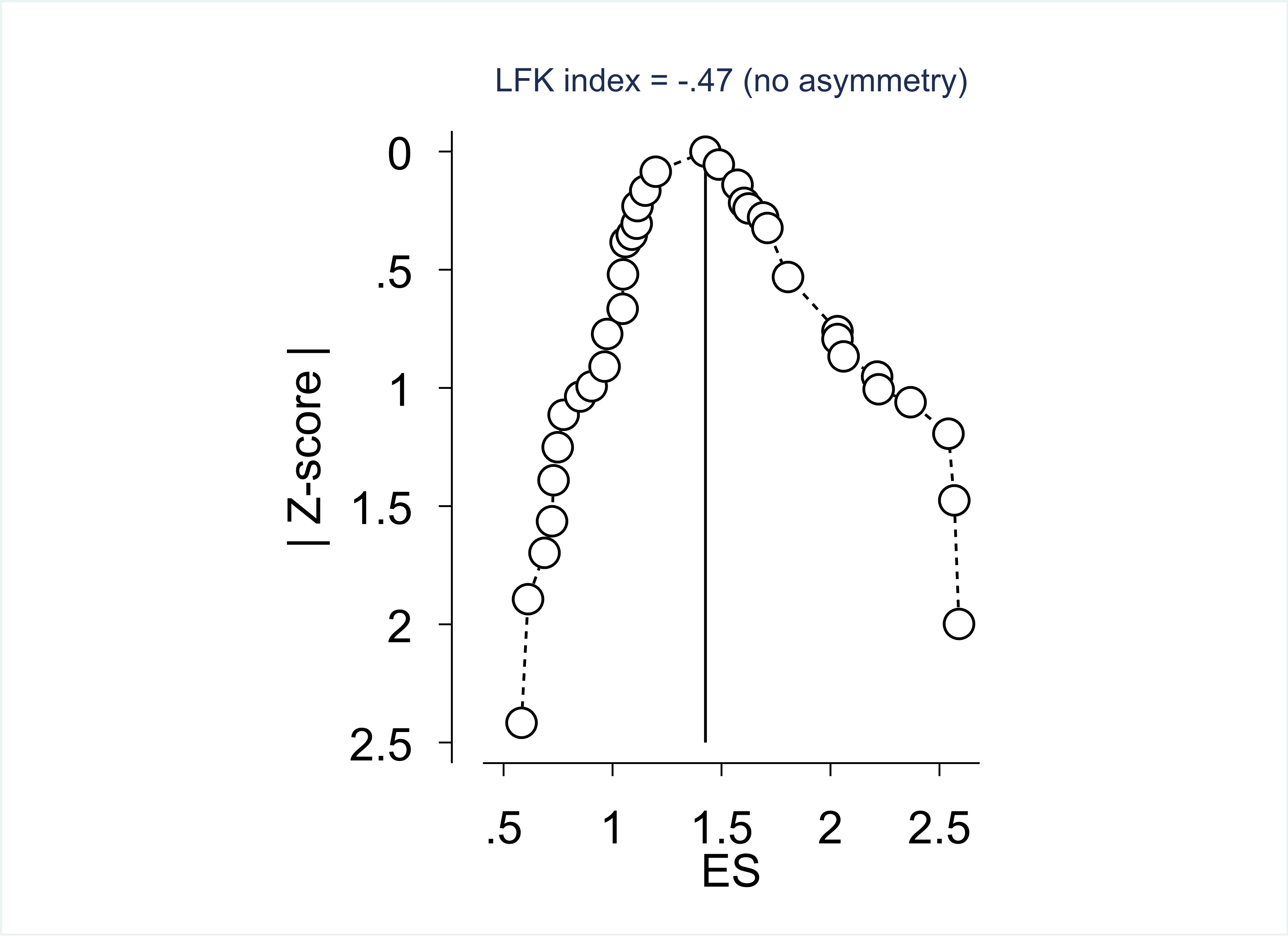

Supplement: S1 Fig — This figure shows the Doi plot used to assess the publication bias. The Doi plot showed no asymmetry suggesting no evidence of publication bias. (PNG) [file pone.0298387.s009.png]
